# Response of submerged macrophytes of different growth forms to multiple sediment remediation measures for hardened sediment

**DOI:** 10.3389/fpls.2024.1450404

**Published:** 2024-09-03

**Authors:** Chuanxin Chao, Xiaorong Chen, Jie Wang, Yonghong Xie

**Affiliations:** ^1^ Key Laboratory of Agro-Ecological Processes in Subtropical Region, Institute of Subtropical Agriculture, Chinese Academy of Sciences, Changsha, Hunan, China; ^2^ Technology Innovation Center for Ecological Conservation and Restoration in Dongting Lake Basin, Ministry of Natural Resources, Changsha, China; ^3^ Dongting Lake Station for Wetland Ecosystem Research, Institute of Subtropical Agriculture, Chinese Academy of Sciences, Changsha, China

**Keywords:** sediment hardening, submerged macrophyte, growth form, loose treatment, litter addition

## Abstract

Climate change and intensified human activities have disrupted the natural hydrological regime and rhythm of river-connected lakes, extending the dry season, increasing water loss, and exposing previously submerged lake floors. This exposure has led to significant sediment hardening, which directly impacts submerged macrophytes. However, strategies to mitigate the negative effects of hardened sediments and promote the growth and development of submerged macrophytes remain largely unexplored. In this study, we selected typical hardened sediment from Dongting Lake to investigate the response of different growth forms of submerged macrophytes to multiple sediment remediation measures (loosening and litter addition) using a mesocosm experiment. The results indicated that loosening alone uniformly benefited all submerged macrophytes by increasing total biomass, relative growth rate (RGR), and the root/shoot ratio. Additionally, loosening altered the root traits of submerged macrophytes, promoting maximum root length (MRL) while reducing average root diameter (ARD). Moreover, different submerged macrophytes exhibited species-specific responses to the combination of loosening and litter addition. Notably, the combination of loosening and adding *Miscanthus lutarioriparius* litter had an antagonistic effect on the growth of *Potamogeton wrightii* and *Myriophyllum spicatum*. The response of functional traits of submerged macrophytes with similar growth forms to the same treatment was consistent. Our findings suggest that future sediment remediation efforts should consider matching specific treatments with the growth forms of submerged macrophytes to achieve optimal outcomes.

## Introduction

Climate change and intensified human activities, such as dam construction, have significantly disrupted the natural hydrological regimes and rhythms of lakes, rendering them some of the most threatened ecosystems globally (Fluet-Chouinard et al., 2023). A recent study has documented a general net decline in lake water storage, revealing a widespread trend of lake drying worldwide (Yao et al., 2023). In river-connected lakes, connectivity with rivers increases the duration of the dry season and degree of water loss. These altered hydrological conditions result in the extended exposure of previously submerged lake floors, leading to extensive sediment hardening. This phenomenon, characterized by the hardening of lake sediments under the current river-lake dynamics, is a novel aspect of lake sedimentology, yet it remains under-researched.

Previous studies have shown a significant decline in submerged macrophytes in lakes worldwide (Sand-Jensen et al., 2000; Zhang et al., 2017). As primary producers, submerged macrophytes are crucial for maintaining the stability of lake ecosystems. Sediment is required for the growth and reproduction of submerged macrophytes with roots (Ozimek, 2006; Verhofstad et al., 2017). Consequently, submerged macrophytes are directly affected by sediment hardening. The phenomenon of soil hardening is a type of soil degradation that has been extensively studied in agricultural ecosystems. Soil hardening is mainly caused by the use of increasingly large and heavy field equipment (Batey, 2009), which differs from the process leading to sediment hardening. However, both forms of hardening have similar effects on the plants that rely on them for growth. In agriculture, soil hardening impairs crop growth and reduces yields (Lipiec and Hatano, 2003; Taylor and Brar, 1991). Similarly, our research indicates that sediment hardening reduces the biomass of submerged macrophytes and shortens their roots, hindering their growth and development (Chao et al., data unpublished). However, effective methods to mitigate the negative effects of hardened sediment on the growth and development of submerged macrophytes remain largely unknown.

Previous studies have shown that one of the most intuitive characteristics of soil hardening is the increased soil stability in terrestrial ecosystems (Candan and Broquen, 2009). This results in higher penetration resistance within the soil profile, limiting root penetration (Blanco-Canqui, 2021). In aquatic ecosystems, the increased stability due to sediment hardening can be detrimental to submerged macrophytes. High stability means that hardened sediment takes longer to return to a loose state after reflooding, which hinders root formation and propagation of submerged macrophytes. Therefore, loosening the hardened sediment may be an important remediation measure to mitigate its negative effects on these plants. Additionally, soil hardening affects not only the physical properties of the soil but also the chemical and biological processes (Das et al., 2023; Hamza and Anderson, 2005). Studies have shown that soil hardening impacts the cycling and mineralization processes of organic matter, carbon, and nitrogen (De Neve and Hofman, 2000; Pandey and Bennett, 2024). Furthermore, soil microbiota can die off in large proportions due to air drying (Haynes, 2000; Kaiser et al., 2015). In this context, the addition of amendments such as biochar is considered a potential biological strategy to help manage soil hardening (Razzaghi et al., 2020). In aquatic ecosystems, litter from hygrophytic plants is abundant, and the decomposition significantly affects the community structure of sediment fauna, organic matter decomposition, sediment properties, and dynamic changes in the carbon cycle of the biological system (Atkinson and Cairns, 2001; Zhou et al., 2023). Therefore, the addition of litter, in combination with sediment loosening, can also be used as a remediation measure to alleviate the negative impact of sediment hardening on submerged macrophytes.

In fact, various sediment remediation measures have altered the sediment environment to some extent (Li et al., 2023). Changes in specific environmental factors can affect particular plant characteristics. For instance, *Vallisneria natans* grown in sandy loam sediment exhibits a reduced root diameter but an increased total root length per plant compared to those growing in clay and mixed sediments (Xie et al., 2005). Additionally, previous studies have shown that the presence of emergent macrophyte detritus in the sediment enhances nutrient content after reflooding and promotes the early growth of *Hydrilla verticillate* (Dainez-Filho et al., 2019). Moreover, different growth forms of submerged macrophytes may have distinct adaptation strategies to changing sediment conditions, displaying species-specific functional traits (Akasaka et al., 2010; Kaijser et al., 2023; Temmink et al., 2021). However, there is a lack of empirical data on how the functional traits of submerged macrophytes with different growth forms respond to multiple sediment remediation measures, such as loosening and litter addition.

Dongting Lake, one of the two major river-connected lakes in the middle reaches of the Yangtze River, is the second-largest freshwater lake in China. Following the operation of the Three Gorges Dam (TGD), the hydrological conditions of Dongting Lake have changed significantly (Geng et al., 2021). The water area of the lake can reach 2670 km^2^ in the flood season and less than 500 km^2^ in the dry season. Influenced by upstream water storage and climate change, a large area of sediment hardening has occurred in Dongting Lake during the dry season. In this study, we selected typical hardened sediment from Dongting Lake to investigate the response of different growth forms of submerged macrophytes to multiple sediment remediation measures (loosening and litter addition) through a mesocosm experiment. The findings of this study provide theoretical support for the remediation and management of sediments in river-connected lakes, and offer a scientific basis for the protection and restoration of submerged macrophytes.

## Materials and methods

### Sediment and plant materials

In May 2023, we collected undisturbed hardened sediments from Dongting Lake. Given that the root depth of submerged macrophytes predominantly extends to 200 mm below the mud surface, we employed PVC tubes with a diameter of 200 mm and a height of 200 mm to acquire intact sediment samples. These sediment cores (200 mm in diameter and 200 mm in height) were then transported to the National Field Scientific Observation and Research Station of the Dongting Lake Wetland Ecosystem for subsequent experiments.

We selected seven submerged macrophytes from Dongting Lake for experimentation. All plant materials were sourced from the seedling pond at the National Field Scientific Observation and Research Station of the Dongting Lake Wetland Ecosystem. One week before the experiment, seedlings of similar size and height were selected for pre-cultivation for each species. The plant height and biomass of all plants were measured at the beginning of the experiment. Based on their growth forms in shallow freshwater lakes, these seven plant species were categorized into three growth forms as referenced in previous literature (Chambers, 1987; Zhao et al., 2022). *Vallisneria natans* and *Chara* spp. were classified into the bottom-dwelling group. *V. natans* is a bottom-dwelling species with stolons and basal leaves, generally reaching heights of 20-100 cm, and is widely distributed in shallow subtropical lakes. *Chara* is macroscopic, with creeping rhizoidal branches from which arise erect branches of limited growth to form low-growing meadows to reduce the resuspension of sediments; *Potamogeton maackianus* and *Stuckenia pectinate* were classified into the erect group. *P. maackianus* and *S. pectinate* are erect species with upright stems and biomass distributed roughly evenly along its length, and both of them exceeded the height of *V. natans* and *Chara*; and *Hydrilla verticillate*, *Myriophyllum spicatum*, and *Potamogeton wrightii* were classified into the canopy-producing group (Zhao et al., 2022). *H. verticillate*, *M. spicatum*, and *P. wrightii* are canopy-producing species with numerous branches and rapid growth, with biomass concentrated near the water surface to form a canopy. Additionally, the amendments chosen were the litter of *Carex brevicuspis* and *Miscanthus lutarioriparius*, both of which are widely distributed in Dongting Lake. On June 1, 2023, 28 plants of each submerged macrophyte, selected for their similar morphology and size, were cultivated. Prior to the experiment, the initial biomass of each submerged macrophyte was measured.

### Experimental set-up

**Figure 1:**
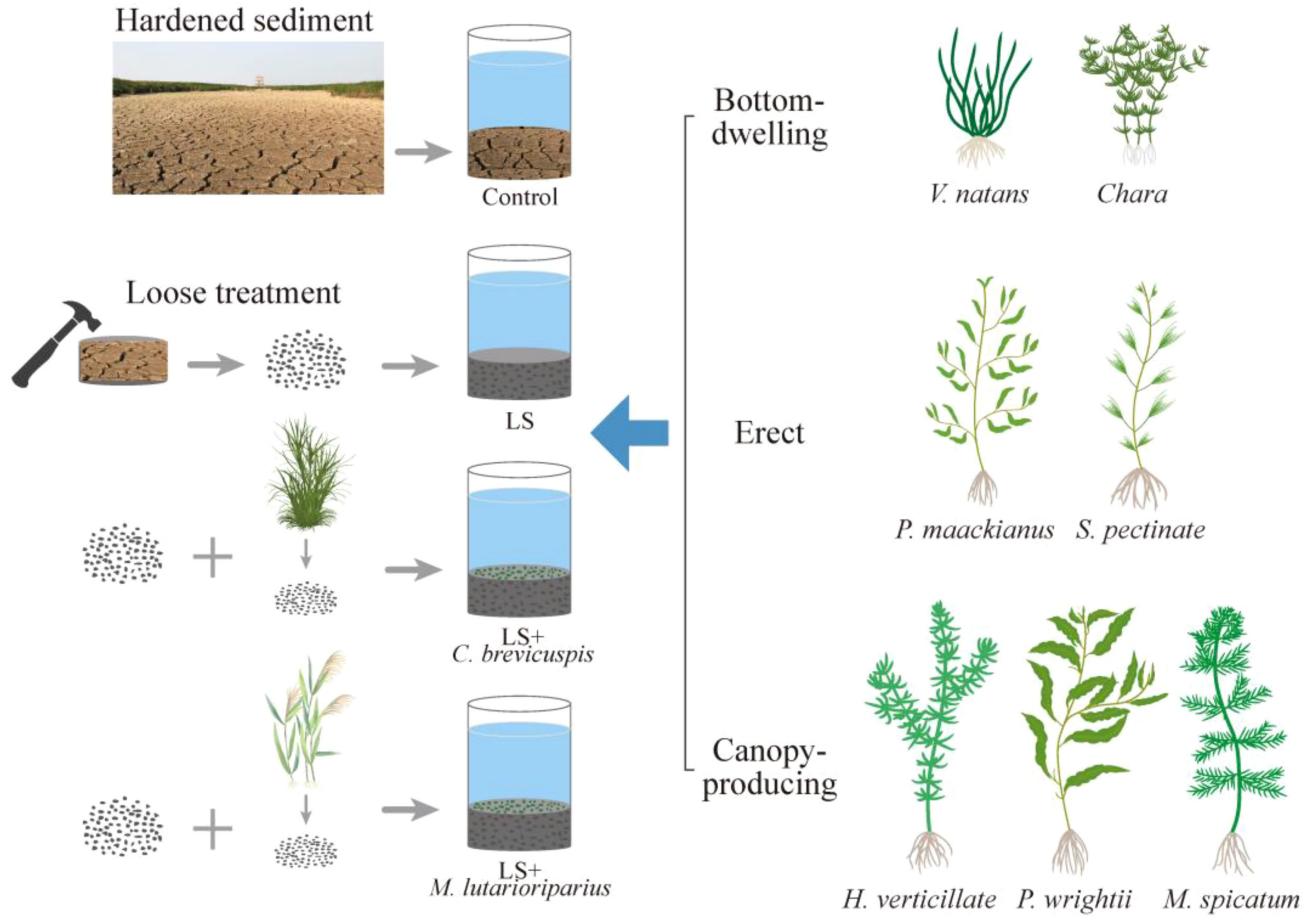
Schematic diagram of experimental design. Control represents sediment cores were hardened and left intact; LS represents sediment cores with hardened sediment were manually crushed, mixed, and reloaded into the tube; LS + *C. brevicuspis* represents sediment cores were first loosened, then *C. brevicuspis* litter was manually crushed, mixed with the loosened sediment, and repacked; LS + *M. lutarioriparius* represents sediment cores were first loosened, then *M. lutarioriparius* litter were added following the same procedure. Seven plant species were categorized into three growth forms (bottom-dwelling group, erect group and canopy-producing group) and planted in the center of each sediment core in each treatment. Each treatment had four replicates (n = 4).

The experiment was conducted from June 8, 2023, to September 8, 2023 (**Figure 1**). We randomly selected 112 sediment cores (TC: 4.08 mg g ^-1^, TN: 2.47 mg g ^-1^, TP: 0.73 mg g ^-1^) from Dongting Lake, with 28 sediment cores allocated to each of the four treatments: (i) Control: sediment cores were hardened and left intact; (ii) Loose Sediment (LS): sediment cores with hardened sediment were manually crushed, mixed, and reloaded into the tube; (iii) LS + *C. brevicuspis*: sediment cores were first loosened, then *C. brevicuspis* litter (Our field survey results suggest that the surface of a sediment core contains approximately 200 g of dry litter; therefore, 200 g of litter debris were added to each sediment core) was manually crushed, mixed with the loosened sediment, and repacked; (iv) LS + *M. lutarioriparius*: sediment cores were first loosened, then *M. lutarioriparius* litter debris (the same amount as *C. brevicuspis*) were added following the same procedure. Each treatment had four replicates, and the sediment cores with different treatments/replicates were randomly placed in the experimental platform’s cement pools (2m × 2m). Eight sediment cores were placed in each cement pool, using a total of 14 cement pools. The cement pool is located outdoors but has a transparent canopy with no shade, with an average water temperature of 29 ± 3.57°C. One submerged macrophyte was planted in the center of each sediment core, with seven species of submerged macrophytes planted in each treatment. Following planting, water (TN: 0.478mg L^-1^, TP: 0.001mg L^-1^) was added to the cement pools and replenish the evaporated water every three days to maintain a water depth of 70 cm.

### Sample collection and functional traits measurements

The experiment concluded after 95 days. Measurements were taken for plant height, ramet number, and maximum root length (MRL). The relative growth rate (RGR) was calculated using the following equation: RGR = (In *w_2_* − In*w_1_*)/(*t_2_* − *t_1_*), where *w_1_* is the initial dry mass at time *t_1_*, *w_2_* is the dry mass at harvest time *t_2_*, and (*t_2_* − *t_1_*) is the experimental duration. After harvesting, the entire root system of each plant was carefully excavated, cleaned with tap water, and transported to the laboratory for further analysis. Root imaging was performed using a root scanner (Epson Perfection v700 Photo), and indices such as the average root length (ARL) and average root diameter (ARD) were obtained with analysis software (WinRHIZO Pro2009a; Regant Instruments Inc., Quebec, Canada). Finally, the plants were dried to constant weight at 75°C and the biomass was measured. The dry weight of the part above the sediment surface represents the shoot biomass, the dry weight of the part below the sediment surface represents the root biomass, and the sum of the two represents the total biomass. The root/shoot ratio is defined as the ratio of root biomass to shoot biomass. Specially, *Chara* grew close to the sediment but did not have a true root system, so the root data were blank in subsequent analyses.

### Statistical analysis

We employed a two-way analysis of variance (ANOVA) to assess the effects of treatments and plant species on the growth characteristics and root functional traits of submerged macrophytes. Prior to data analysis, we performed log_10_, square root arcsine, and Box-Cox transformations on data that did not meet the assumptions of variance normality and homogeneity. Differences in growth characteristics and root functional traits among species within each treatment were evaluated using Tukey’s test for multiple comparisons. Specifically, the seven plants were categorized into three growth forms, and eight functional traits were analyzed as their attributes. Nonmetric multidimensional scaling analysis (NMDS) was utilized to evaluate the variations among different growth forms across treatments, while permutation multivariate analysis of variance (PERMANOVA) was used to test the significance of differences between these groups. Both NMDS and PERMANOVA were conducted using the ‘vegan’ package in R (Oksanen et al., 2016). All computations were performed using R (version 3.6.3) (R Development Core Team, 2019).

## Results

### Effects of multiple sediment remediation measures on submerged macrophytes growth and biomass allocation

**Figure 2:**
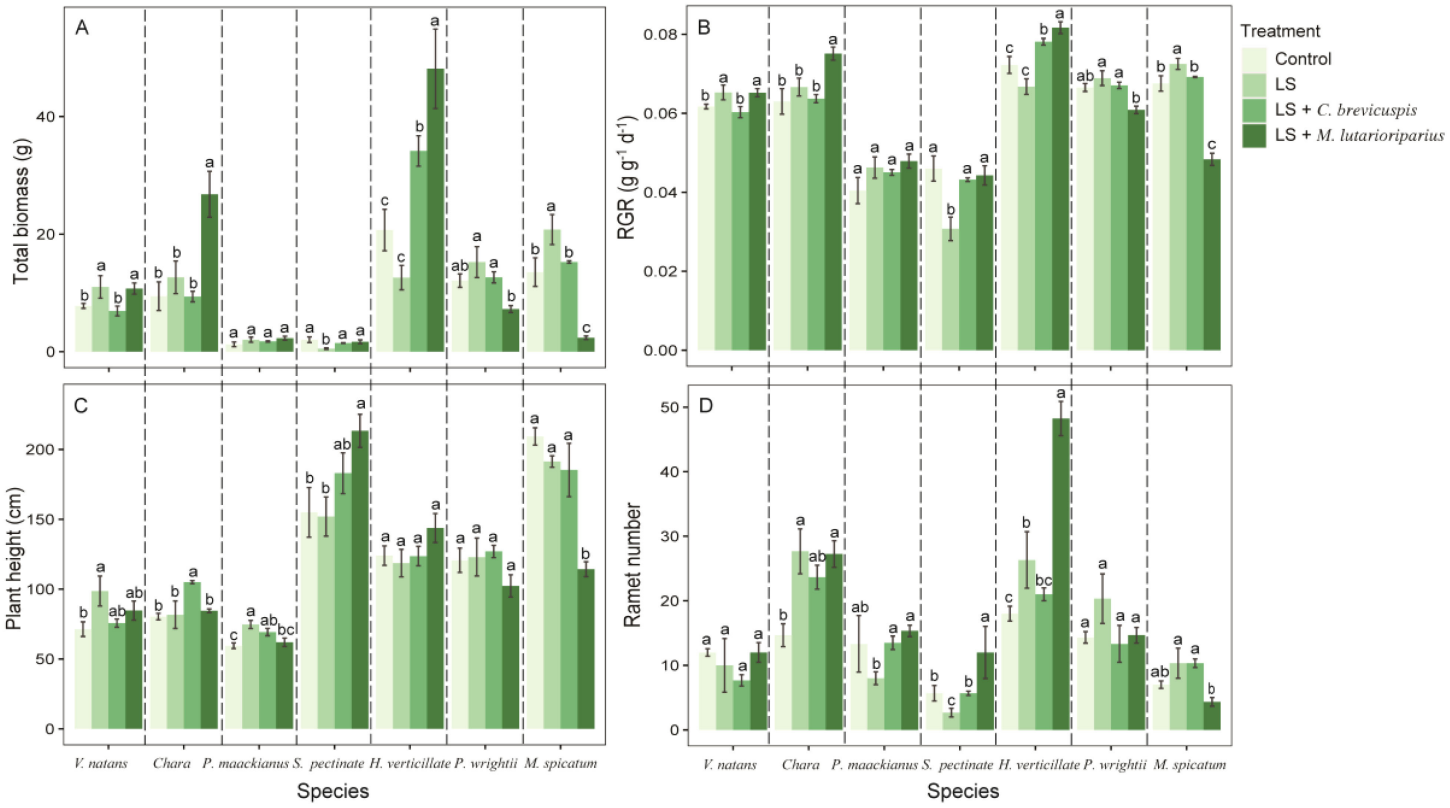
Effects of different treatment on measures of functional traits of different species. **(A)** total biomass, **(B)** relative growth rate, **(C)** plant height and **(D)** ramet number. Data shown are the mean ± SE, n = 4. We have separated the different species by dotted lines. Different lowercase letters between dotted lines indicated significant difference between treatments for the same species (*p* < 0.05).

**Figure 3:**
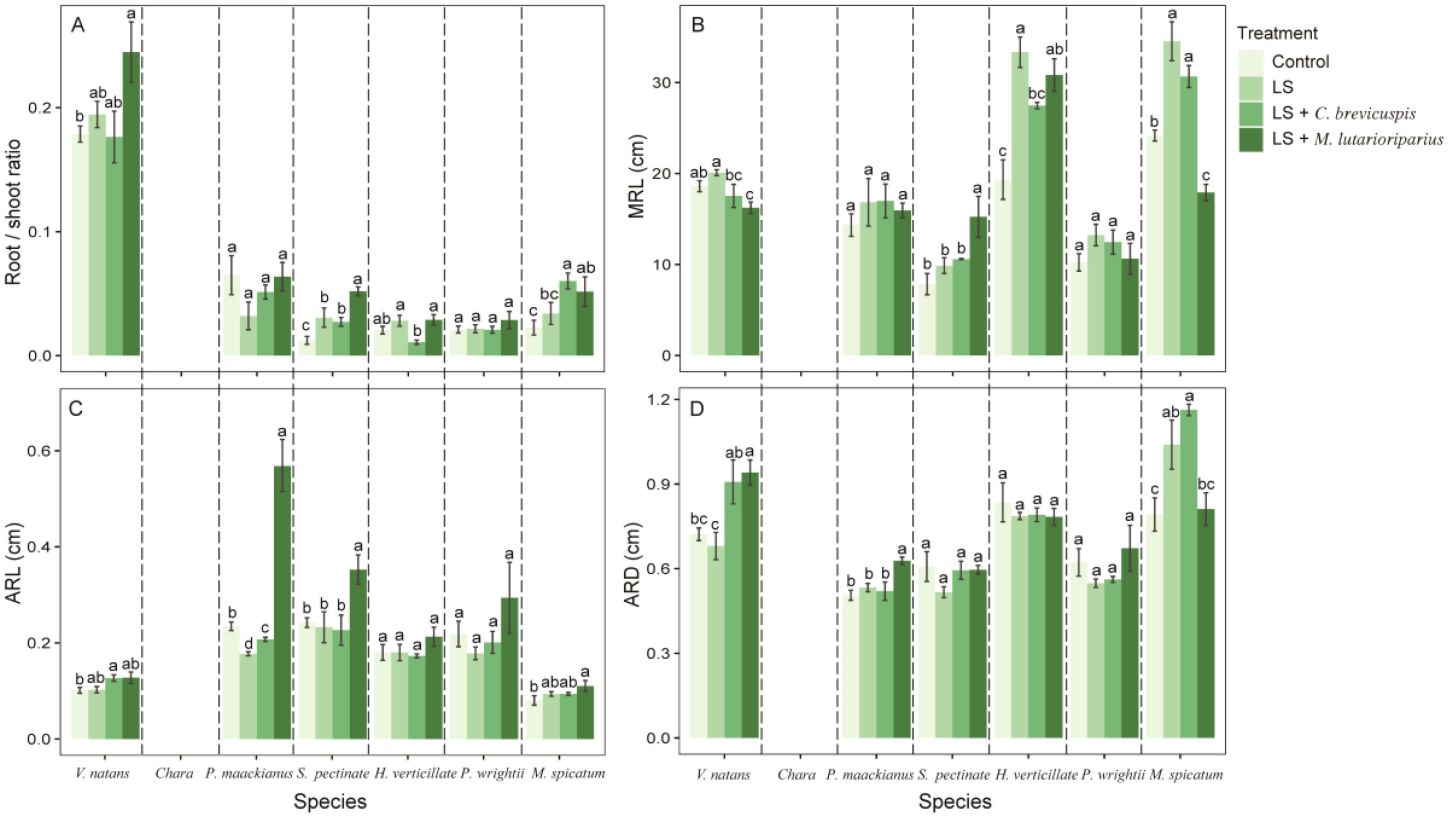
Effects of different treatment on measures of functional traits of different species. **(A)** root:shoot ratio, **(B)** maximum root length, **(C)** average root length and **(D)** average root diameter. Data shown are the mean ± SE, n = 4. We have separated the different species by dotted lines. Different lowercase letters between dotted lines indicated significant difference between treatments for the same species (*p* < 0.05).

Regarding the growth morphology and biomass allocation of seven submerged macrophytes, different experimental treatments had significant overall effects on total biomass, RGR, ramet number, and root/shoot ratio (**Table 1**). Compared with the control group (hardened sediment), loosening alone (LS) increased the total biomass and RGR of submerged macrophytes, except for *S. pectinata* and *H. verticillata* (**Figures 2A, B**). For ramet number and root/shoot ratio, loosening alone increased the ramet number of *Chara*, *H. verticillata*, *M. spicatum*, and *P. wrightii*, but reduced the root/shoot ratio of *P. maackianus* (**Figures 2D**, **3A**). Additionally, loosening alone significantly increased the plant height of *V. natans* and *P. maackianus* (**Figure 2C**).

**Table 1:** *F-*value and significance of the two-way analysis of variance (ANOVA) results for the effects of treatment and species on measures of functional traits of submerged macrophytes.

	Treatment		Species		Treatment*Species	
	*F*	*P*	*F*	*P*	*F*	*P*
Total biomass	2.777	**0.049**	183.766	**< 0.001**	12.997	**< 0.001**
Root/shoot ratio	7.463	**< 0.001**	78.26	**< 0.001**	3.623	**< 0.001**
RGR	3.898	**0.013**	180.07	**< 0.001**	14.284	**< 0.001**
Ramet number	7.961	**< 0.001**	50.289	**< 0.001**	5.192	**< 0.001**
Plant height	1.08	0.364	97.361	**< 0.001**	4.052	**< 0.001**
Maximum root length	9.474	**< 0.001**	78.644	**< 0.001**	4.043	**< 0.001**
Average root length	19.505	**< 0.001**	66.887	**< 0.001**	2.805	**0.003**
Average root diameter	3.742	**0.017**	53.949	**< 0.001**	3.781	**< 0.001**

*P*-values less than 0.05 are shown in bold.

Furthermore, the LS + *C. brevicuspis* treatment significantly increased the total biomass and RGR of *H. verticillata* compared to the control group (**Figures 2A, B**). This treatment also significantly augmented the plant height of *Chara* and *P. maackianus* and increased the root/shoot ratio of *S. pectinata* and *P. wrightii* (**Figures 2C**, **3A**). In addition, the LS + *M. lutarioriparius* treatment significantly increased the total biomass and RGR of *V. natans*, *Chara*, and *H. verticillata* compared to the control group, but decreased the total biomass and RGR of *M. spicatum* and *P. wrightii* (**Figures 2A, B**). For ramet number and root/shoot ratio, this treatment significantly increased the ramet number of *Chara*, *S. pectinata*, and *H. verticillata*, as well as the root/shoot ratio of *V. natans* and *S. pectinata* (**Figures 2D**, **3A**). However, compared to the control group, the LS + *M. lutarioriparius* treatment significantly augmented the plant height of *S. pectinata* but reduced the plant height of *P. wrightii* (**Figure 2C**).

### Effects of multiple sediment remediation measures on root traits of submerged macrophytes

Our results shown that different experimental treatments significantly influenced the root traits of submerged macrophytes (**Table 1**). Compared to the control group, loosening alone increased the MRL of submerged macrophytes but reduced the ARD, except for *P. maackianus* and *P. wrightii* (**Figures 3B, D**). However, different combinations of remediation measures had varying effects on the root traits of submerged macrophytes. Compared to the control group, the LS + *C. brevicuspis* treatment significantly increased the MRL of *H. verticillata* and *P. wrightii* (**Figure 3B**). On the other hand, the LS + *M. lutarioriparius* treatment significantly increased the MRL of *S. pectinata* and *H. verticillata* but reduced the MRL of *V. natans* and *P. wrightii* (**Figure 3B**). For ARL and ARD, compared to the control group, the LS + *C. brevicuspis* treatment significantly increased the ARL and ARD of *V. natans*, while the LS + *M. lutarioriparius* treatment significantly increased the ARL of *P. maackianus*, *S. pectinata*, and *P. wrightii*, and the ARD of *V. natans* and *P. maackianus* (**Figures 3C, D**).

### Growth forms response to treatments

**Figure 4:**
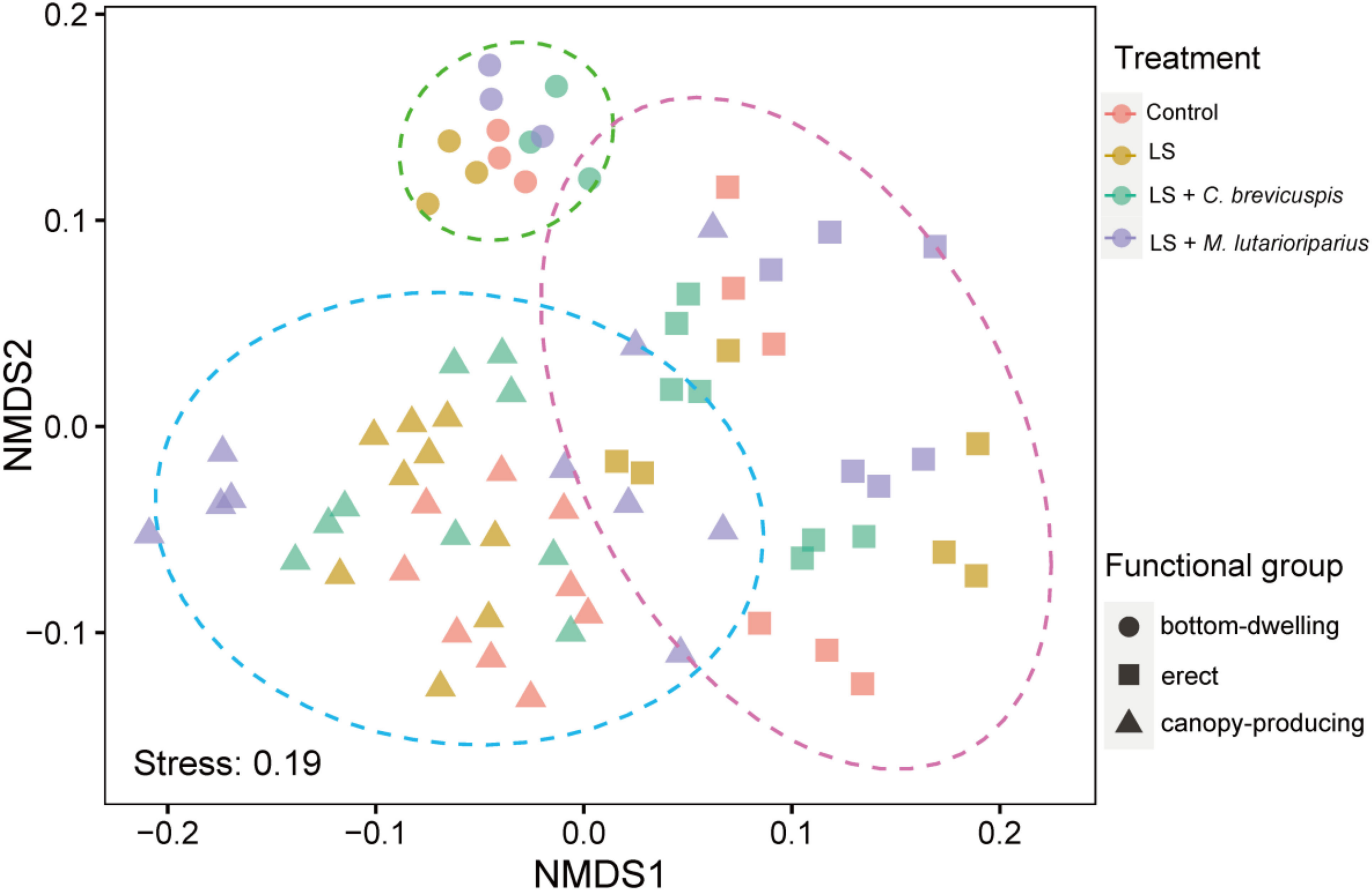
Nonmetric multidimensional scaling diagram showing submerged macrophytes functional traits differences over different growth forms and treatment.

In our study, seven submerged macrophytes were categorized into three growth forms: bottom-dwelling, erect, and canopy-producing. To comprehensively evaluate the differential responses of these growth forms to various treatments, we considered eight functional traits (total biomass, RGR, plant height, ramet number, root/shoot ratio, MRL, ARL, and ARD) as attributes of each growth form and employed NMDS for further analysis. The NMDS analysis revealed a distinct clustering of functional indicators according to the different growth forms across the four treatments (**Figure 4**). The results of PERMANOVA indicated that functional traits were significantly affected by growth forms but not by treatments, and the interactive influences between them were not significant (**Table 2**).

**Table 2:** The effects of growth forms and treatment on functional traits based on PERMANOVA.

	*Df*	*F*	*R^2^*	*P*
growth forms	2	13.33	0.28	**0.001**
Treatment	3	0.40	0.01	0.898
growth forms× Treatment	6	0.76	0.05	0.663

Significant effects (*P* < 0.05) are indicated in bold.

## Discussion

Sediment provides a fundamental substrate for submerged macrophytes to anchor, reproduce, and grow steadily, serving as a direct nutrient source (Barko and Smart, 1986). Under the interaction between rivers and lakes, sediment hardening has emerged as a new characteristic of lake sediment. Compared to non-hardened sediment, hardened sediment reduces the biomass of submerged macrophytes and shortens their root systems, hindering their growth and development (Chao et al., data unpublished). In this study, our results demonstrated that loosening alone increased the total biomass and relative growth rate (RGR) of submerged macrophytes compared to the control group, although the increase was not significant for few species. Previous studies have indicated that soil air drying increases the stability of soil aggregates (Kaiser et al., 2015), and the degree of sediment hardening is closely linked to aggregate stability (Candan and Broquen, 2009). These suggests that physical loosening of hardened sediments benefits the growth of submerged macrophytes, likely due to the reduced stability of soil aggregates. Furthermore, asexual propagation is the primary mode of reproduction for submerged macrophytes, with sediment playing a crucial role in the propagation and colonization of propagules (Philbrick and Les, 1996). Thus, in our study, loosening the sediment disrupted its high stability, facilitating the growth of submerged macrophytes. Additionally, the increase in biomass and RGR may be attributed to enhanced nutrient uptake by submerged macrophytes in loosened sediments. The soil air drying process increases macroaggregates (>0.25 mm) (Kaiser et al., 2015), and the protective environment formed by macroaggregates prevents the contact of nutrients in them with the outside world (Huang et al., 2017), which may not be conducive to the absorption of submerged plant roots. Therefore, the physical alteration of sediment through loosening is more conducive to the expansion and high biomass accumulation of submerged macrophytes.

Additionally, our results showed that loosening alone increases the root/shoot ratio of submerged macrophytes, indicating that these plants allocate more biomass to their roots. Previous studies have demonstrated that biomass allocation patterns are crucial ecological strategies for submerged macrophytes to adapt to environmental changes (Chen et al., 2020; Liu et al., 2021). The failure of root anchorage in lake sediments is a significant factor contributing to the decline of submerged macrophyte communities and impeding their recovery (Schutten et al., 2005). We infer that the increased biomass allocation to roots under loosening treatment helps submerged macrophytes anchor more effectively in the sediment, counteracting the buoyancy forces exerted by water, and thereby stabilizing the colony. Conversely, the relatively low root/shoot ratio of submerged macrophytes in hardened sediment suggests a reduced ability to withstand the forces of waves and currents.

However, the response of submerged macrophytes’ growth performance and biomass allocation to combined sediment remediation measures was inconsistent. The decomposition of macrophytes in sediments affects carbon and nutrient cycling as well as energy flow, with high-quality litter (e.g., high initial nitrogen and phosphorus content) typically decomposing faster than low-quality litter (Grasset et al., 2019; Wang et al., 2017). Additionally, different species of submerged macrophytes exhibit varying nutrient absorption efficiencies (Lu et al., 2018). These studies suggest that the LS + *C. brevicuspis* and LS + *M. lutarioriparius* treatments do not uniformly stimulate all submerged macrophytes. We found that LS + *C. brevicuspis* group significantly increased the total biomass and RGR of *H. verticillate* compared to the control group and loose treatment group, while LS + *M. lutarioriparius* treatment significantly increased the total biomass and RGR of *V. natans*, *Chara* and *H. verticillate*. Our results further suggest that submerged macrophytes exhibit species-specific responses to multiple remediation measures. Notably, the LS + *M. lutarioriparius* treatment significantly reduced the biomass and RGR of *P. wrightii* and *M. spicatum*. From a practical application perspective, our findings suggest that combining *M. lutarioriparius* addition with sediment loosening has an antagonistic effect on the growth of *P. wrightii* and *M. spicatum*. This may be due to the accumulation of toxic soluble organic carbon compounds during anaerobic decomposition (Barko and Smart, 1983). Previous studies have suggested that the positive effects of detritus decomposition on submerged macrophytes may be counteracted by negative effects over a longer decomposition period (Dainez-Filho et al., 2019). Furthermore, we found that the LS + *M. lutarioriparius* treatment had a greater effect on the root/shoot ratio of submerged macrophytes compared to the LS + *C. brevicuspis* treatment. For example, the LS + *M. lutarioriparius* treatment significantly increased the root/shoot ratio of *V. natans* and *S. pectinate* compared to the LS + *C. brevicuspis* and loose treatments. This may be because *M. lutarioriparius* litter decomposes faster than *C. brevicuspis* litter, causing submerged macrophytes to allocate more biomass belowground to develop roots that efficiently absorb the released nutrients (Zhu et al., 2021, 2022).

Resource acquisition is one of the main functions of roots, and variations in root traits reflect the strategies plants use to obtain resources effectively (Tajima, 2021). Our results showed that loosening alone increased the maximum root length (MRL) of submerged macrophytes but reduced the average root diameter (ARD), except for *P. maackianus* and *P. wrightii*. Previous studies have demonstrated that integrating root traits enables plants to adapt to different environments, resulting in higher productivity (Moler et al., 2022). Plants with long, thin roots are more efficient in nutrient acquisition than those with short, thick roots (Xie et al., 2006, 2005). Root development determines root distribution, and the relatively large MRL and small ARD in the loosening treatment group indicated a wider range of resources available to submerged macrophytes and higher efficiency. This also reflected that sediment hardening limited the ability of submerged macrophyte roots to absorb nutrients. Furthermore, we found that combining multiple remediation measures could significantly increase the ARD of some submerged macrophytes (e.g., *V. natans* and *P. maackianus*). Notably, the LS + *M. lutarioriparius* treatment shortened the MRL of *V. natans* and *M. spicatum*. Previous studies have shown that root traits exhibit plasticity in response to different sediment types and nutrient conditions (Li et al., 2011; Pan et al., 2012). These results suggest that the effects of combined remediation measures on the root traits of submerged macrophytes are not uniform, showing species-specific responses.

Submerged macrophytes of different growth forms have distinct ecological niches and functional traits in aquatic ecosystems (Liu et al., 2021; Manolaki et al., 2020). Our study showed that functional traits under different treatments were clearly differentiated by growth forms, indicating that the response of functional traits within the same growth form to treatment is similar. Functional traits determine plant growth and development and influence their ecological functions (Tao et al., 2024; Zhao et al., 2022). To a certain extent, growth forms reflect the adaptation strategies of submerged macrophytes to their habitats (Chambers, 1987). For example, under low-light conditions, different growth forms of submerged macrophytes exhibit varying changes in functional traits. Bottom-dwelling *V. natans* has characteristics of low light compensation and low light saturation points, producing more belowground biomass through stolon extension to adapt to unfavorable environments (Fu et al., 2012). In contrast, canopy-producing *M. spicatum* adapts by increasing aboveground biomass to produce more branches (Tao et al., 2024). In our study, multiple sediment remediation measures created different sediment conditions. From a practical application perspective, understanding the likely changes in functional traits at the growth form level can guide us in associating specific treatments with particular classes of submerged macrophytes to achieve optimal outcomes.

In conclusion, sediment hardening, a new feature of lake sediments, restricts the development of submerged macrophytes. By applying multiple sediment remediation measures, the negative effects of sediment hardening can be alleviated to some extent, promoting the growth and development of submerged plants. Loosening hardened sediment can increase biomass accumulation and enhance root development, generally benefiting various submerged macrophytes. The functional traits of submerged macrophytes showed species-specific responses to the combination of loosening treatment and litter addition. Functional traits of submerged macrophytes with the same growth form responded similarly to the same treatment. When implementing multiple sediment remediation measures in the future, treatments can be tailored to specific growth forms of submerged macrophytes to achieve the most effective results.

## Data Availability

The original contributions presented in the study are included in the article/supplementary material. Further inquiries can be directed to the corresponding author.
